# Factors influencing utilisation of cervical cancer screening services among HIV positive women attending care and treatment centres in Kinondoni municipality, Dar es Salaam, Tanzania

**DOI:** 10.4314/ahs.v24i2.5

**Published:** 2024-06

**Authors:** Eliena Kisaka, Titus Kabalimu, Innocent Semali, Yohana Mashalla

**Affiliations:** 1 Hubert Kairuki Memorial University Faculty of Medicine, Community Medicine; 2 Hubert Kairuki Memorial University, Department of Physiology

**Keywords:** Cervical cancer, HIV positive women

## Abstract

**Background:**

Cervical cancer is among the leading causes of cancer-related deaths among HIV+ve women.

**Objective:**

To determine factors influencing utilisation of cervical cancer screening among HIV+ve women attending Cancer Treatment and Care in Kinondoni Municipality, Dar es Salaam.

**Methods:**

Cross-sectional study among HIV+ve women was carried out between September and October 2021; collected using a standardised questionnaire. Descriptive statistics, bivariate and multivariate analyses were used to determine cervical cancer extent and association of predictors of cervical cancer screening.

**Results:**

230 HIV+ve women aged 21–60 years were interviewed. Only 47% had screened for cervical cancer. Low knowledge of HIV+ve as risk significantly associated with less likelihood to screen for cervical cancer [AOR 0.49, 95% CI (0.253-0.957, P = 0.037)]. Parity of 3 or more was twice likely to screen for cervical cancer [AOR 2.124, 95% CI (1.012-4.456, P = 0.046)]; and housewives were 2.5 more likely to screen for cervical cancer [AOR 2.594, 95% CI (1.149-5.853, P = 0.002)]. Lack of knowledge on preventive measures was less associated with likelihood to screen [AOR 0.114, 95% CI (0.013-0.972, P = 0.047)].

**Conclusion:**

Lack of knowledge on HIV+ve and prevention, age and parity are likely to influence utilisation of cervical cancer screening services.

## Introduction

While cervical cancer is preventable, it remains a major public health problem in the many countries. In 2013, there were 485,000 cervical cancer cases and 236,000 women deaths were due to cervical cancer worldwide.^1^ Cervical cancer accounted for 87% of deaths; was the second most commonly diagnosed cancer and third leading cause of cancer death among females in less developed countries.^2^ In 2020, it was estimated that 604,237 women were diagnosed with cervical cancer representing 6.5% of all female cancers.^3^ Deaths due to cervical cancer were estimated at 341,843 of whom 90% were reported in less-developed regions of the world where access to prevention, screening, and treatment services are limited.^3^ East Africa has the highest sub-regional incidence of cervical cancer of 42.7 per 100,000 women followed by Southern Africa region with 31.5 per 100,000 women.^4^ Tanzania has a high burden of cervical cancer as recently indicated that 10,241 new cervical cancer cases were reported in 2020 and the number of deaths were 6,525 accounting for 63.7% of all adult female deaths.^5^ It has been reported that 5% of all cervical cancer cases are attributed to HIV.^6^ The attribution proportion in sub-Saharan Africa is high, estimated that 63.8% of the patients with cervical cancer are living with HIV^7^. The interactive effect of cervical cancer risk factors with HIV account for the high burden of cervical cancer in most sub-Saharan countries.^8^

The global response calls for cervical cancer elimination through 70-90-90 by 2030 and the World Health Organisation (WHO) has developed guidance towards prevention and control of cervical cancer through vaccination, screening services, management of invasive cancer, and knowledge repository.^9^,^10^ Studies in East Africa have shown that while comprehensive vaccination is cost-effective and lifesaving, the incidence of cervical cancer is not expected to drop for at least two decades after widespread vaccination uptake.^11^ The success of screening depends on factors such as access, uptake and treatment for those who screen positive. According to WHO, women aged 30 years should start screening for cervical cancer^12^ and screening at least once in a lifetime is beneficial, and intervals may depend on existing infrastructure and resources. Decisions on the frequency of screening and target ages are determined by costs, existing burden of disease and infrastructure.^13^ Cytology-based screening is not practical for wide-spread use in sub-Saharan Africa due to high cost, low sensitivity, inherent need for elaborate laboratories, trained technicians and complex follow-up protocols.^14^. Testing for Human papillomavirus (HPV) is higly recommended as the primary screening modality where feasible.^15^,^16^ Hence, HPV-DNA testing is the most objective and sensitive screening approach which has shown to decrease mortality from cervical cancer in low-resource settings.^13^,^17^-^19^ Visual inspection with acetic acid (VIA) is an acceptable alternative where HPV testing is cost-prohibitive.^15^,^16^

The escalating cervical cancer burden in sub-Saharan Africa is caused by inadequate resources to provide required services leading to limited access to cervical cancer screening, delays in reporting at health facilities, low level of awareness and prohibitive cultures to women from accessing health services.^20^,^21^

Tanzania has aligned to the global efforts through the Strategic Development Goals frameworks and local operationalisation 2030.^22^ This includes among others, integration of cervical screening and care services with HIV care and treatment services. The National Guideline for the management of HIV/AIDS recommends that annual cervical cancer screening using VIA or rapid HPV testing as the primary screening method should be integrated into the national policy as part of routine care for HIV-positive women. Care and Treatment Centres (CTC) should be closely linked with centres that provide services for cervical cancer prevention or the centres should develop capacities to provide such services. In addition, cervical cancer screening should be done at HIV diagnosis and repeat annually regardless of previous results among sexually active girls and women.^23^ However, despite the integration and roll out, its implementation has not been fully evaluated. This study aims to assess the factors that have influenced utilisation of cervical cancer screening among HIV+ve women attending Cancer Treatment and Care in Kinondoni Municipality, Dar es Salaam.

## Methods

### Study design and setting

The Dar es Salaam region has five municipalities including Ilala, Temeke, Ubungo, Kinondoni, and Kigamboni. We conducted a population-based cross-sectional study in the Kinondoni Municipality between September and October 2021. Kinondoni Municipality has a total area of 321km^2^ with population estimated at 1,134,211.^24^ There are 24 hospitals, 9 health centres and 97 dispensaries in the Kinondoni Municipality of which 23 hospitals, 7 health centres and 74 dispensaries are privately owned and 1 hospital, 2 health centres and 23 dispensaries are public health facilities. Cervical cancer prevention interventions have been implemented in Dar es Salaam region and the target population of this study was HIV positive women aged 21 years and above attending Care and Treatment Centre services.

### Sampling procedures and sample size

The Kinondoni Municipal was randomly selected from the five Dar es Salaam municipalities. The sampling frame was all hospitals and health centres providing CTC services in Kinondoni district. From the sapling frame, simple random sampling was used to select six health facilities by using a lottery method of allocating numbers from which the facilities were randomly selected.

The patients were HIV positive women who had been receiving services at the CTC clinics for at least a month or more and had voluntarily consented to participate in the study. The minimum sample size was determined using Cochran formula^25^ based on the study done among HIV positive women at the Dodoma Regional Referral Hospital at CTC clinic^26^. The prevalence p was 16% and a level of accuracy was set at 5% to allow a 95% interval around estimate. The estimated study sample was 207 with an additional 10% of this number added to make up for non-responses. The minimum sample size was therefore, 230 patients and participants were enrolled from the selected health facilities into the study consecutively till the required sample size was realised.

### Data collection procedure

Data was collected using closed end-pre-coded questionnaires through face-to-face interview. The interviews lasted for 10 – 15 minutes. The questionnaire was prepared in English and translated into Kiswahili by a competent person fluent in both languages. The questionnaire collected data on women's socio-demographics including age, education, marital status, employment and information on sexual practices, parity, knowledge, attitude and perception regarding cervical cancer and screening for cervical cancer. The patient record (Care and Treatment Centre) cards were used to verify collected demographic data of each participant. Interviews were conducted by the Principal Investigator and Research Assistants recruited from the health facilities in Kiswahili the local language spoken in Tanzania. Research assistants were trained on the principles of quantitative research including data collection, objectives of the study and procedures for sampling, interviewing and consent procedures. The outcome variable of the study was cervical cancer screening measured in terms of whether respondents underwent any cervical cancer screening test ever; and the explanatory variables included socio-demographic information including age, education, marital status, parity as well as health characteristics and employment.

### Data management and analysis

Data from the questionnaire was reviewed on daily basis, checked for clarity, completeness and consistency. Meetings with research assistants were held daily to determine the quality and comparability of the data and Dbase IV computer software was used to capture data from the questionnaires. Each variable field was formatted according to the variable type and Statistical Package for Social Sciences (SPSS) version 25 was used to analyse the data. Frequencies and percentages were used to summarise socio-demographic characteristics, individual factors (knowledge, attitude and perception), reproductive and sexual practices factors, family history of cervical cancer and screening. The quantitative independent and some nominal variables were categorised and cross-tabulated with the dependent variables. Chi squared test was used to determine the significance of association between independent variables. Bivariate analyses were performed to determine the association between dependent and independent variable using crude odds ratio (COR). Odds ratios were reported with accompanying 95% confidence intervals. The significance of association was assessed using OR, 95% confidence interval and p-value at < 0.05. The adjusted odds ratios with its 95% CI for associated factors are reported.

### Ethical consideration

The study received ethical clearance from the Hubert Kairuki Memorial University Institutional Ethics Review Board (Ref: HK/IREC/60/01/20) and permission to carry out the study in the health facilities in Kinondoni Municipality were obtained from the Regional Medical Office, Municipal Chief Medical Officer and health facilities management. Meetings with the authorities were held during which the study purpose and methods were explained. Interviews were held in an arranged separate room. The study objectives and the methods were explained to the participants. Participants were informed that the study carried minimal risks and no direct benefits or incentives would be given. Participants were further informed that participation in the study was voluntary and refusal to participate would not have any consequences on their rights to quality services they were receiving. Participants were given time to ask questions and clarification on any issues related to the study. Upon comprehension, each participant was then asked to consent by signing on the consent form.

## Results

Two hundred thirty HIV positive women were interviewed. The mean age was 38.79 years. Table 1 presents the socio-demographic characteristics of the study population. Majority (42.6%) were in the age group 36-45 years; 131(57%) had attended primary education and 6.1% had no formal education. Most participants 152(66.1%) were businesswomen/self-employed, 98(42.6%) were married and 58.7% had parity of 1 or 2.

**Table 1:** Demographic characteristics of the HIV positive women attending Care and Treatment Centres in Kinondoni Municipality, Dar es Salaam, Tanzania

Variable	N	%
**Age group (years)**		
21 - 35	79	33.6
36 - 45	98	41.7
46 - 60	58	24.7
**Education level**		
Primary education	128	54.5
Secondary education	63	26.8
University/college	25	10.6
education		
No formal education	19	8.1
**Employment status**		
Businesswoman	152	64.7
Informal sector	40	17.0
Housewife	43	18.3
**Marital status**		
Married	81	34.5
Single	73	31.1
Missing data	81	34.5
**Parity**		
None	33	14.0
1 - 2 births	135	57.4
3 or more births	67	28.5

### Bivariate analysis results

Table 2 presents logistic bivariate analyses results of the association of the dependent and independent variables using crudes Odds ratio. Less than half (46.9%) had screened for cervical cancer. Cervical cancer screening significantly associated with older ages 46-60 years [COR=1.82, 95%CI (1.26 - 5.25), P = 0.01)]; parity of 3 and above [COR=2.29, 95%CI (1.26 - 4.18), P = 0.007)]; and housewives [COR=2.29, 95%CI (1.04 - 4.18), P = 0.039)]. Less likelihood to screen for cervical cancer significantly associated with lack of knowledge on cervical cancer risk factors [COR=0.41, 95%CI (0.23-0.72), P = 0.002]; no family history of cervical cancer [COR=0.25, 95%CI (0.09-0.69), P = 0.008] and lack of knowledge that being HIV positive is a risk factor for cervical cancer [COR=0.041, 95%CI (0.23-0.72), P = 0.002)]. Education, employment and history of STDs did not significantly associated with cervical cancer screening.

**Table 2:** Bivariate analysis results of factors influencing cervical cancer screening among HIV positive women attending Care and Treatment Centres in Kinondoni Municipality, Dar es Salaam, Tanzania

Variable	N	Screened (%)	COR*	95% CI		p-Value
**Age years**						
21 - 35	79	33.6	1			
36 - 45	98	41.7	1.821	0.99	3.345	0.053
46 - 60	58	24.7	1.821	1.26	5.246	0.01
**Education level**						
Primary education	128	54.5	1			
Secondary education	63	26.8	0.856	0.46	1.581	0.621
University/college education	25	10.6	0.966	0.41	2.275	0.938
No formal education	19	8.1	0.582	0.19	1.829	0.354
**Parity**						
1 - 2 births	135	40.7	1			
3 and more births	67	61.2	2.294	1.26	4.177	0.007
None	33	14.0	1.091	0.48	2.485	0.836
**Employment status**						
Businesswoman	152	44.7	1			
Informal sector	40	38.2	0.765	0.36	1.638	0.49
Housewife	43	62.8	2.084	1.04	4.182	0.0395
**Age at sex onset**						
10 - 19 years	17	47.1	1			
20 - 25 years	213	46.8	0.96	0.84	1.101	0.559
**Knowledge on risk factors**						
Yes	31	71	1			
No	199	43.2	0.405	0.23	0.719	0.002
**Family history of cervical cancer**						
Yes	21	76.2	1			
No	209	44	0.246	0.09	0.696	0.008
**Knowledge of HIV+ve as risk factor**						
Yes	153	54.2	1			
No	77	32.5	0.405	0.23	0.719	0.002
**Previous history of STDs**						
Yes	17	58.8	1			
No	213	46	0.596	0.22	1.656	0.313

### Multivariate analysis results

The multivariate analyses results (Table 3) show that women who have no knowledge of HIV as a risk factor for cervical cancer had higher Odds (51%) of not undergoing screening [AOR 0.49, 95% CI (0.253 - 0.957), P = 0.037)] while women whose parity was 3 and above had more than twice likelihood of being screened for cervical cancer [AOR 2.124, 95% CI (1.012 - 4.456), P = 0.046)]. When compared to businesswomen, housewives were about 2.5 more likely to screen for cervical cancer [AOR 2.594, 95% CI (1.149 - 5.853), P = 0.02)] and women who had no knowledge on preventive methods were less likely to screen for cervical cancer [AOR 0.114, 95% CI (0.013 - 0.972), P = 0.047)]. Age at onset of sex and history of STD did not significantly associate with cervical cancer screening.

**Table 3:** Association between demographic characteristics and screening for cervical cancer among HIV positive women attending Care and Treatment Centres in Kinondoni Municipality, Dar es Salaam, Tanzania

Variable	N	Adjusted Odds Ratios	95% Confidence Interval		p-value
**Age group (years)**					
21-35	79	1			
36-45	98	1.112	0.53	2.33	0.78
46-60	58	1.525	0.598	3.89	0.377
**Education level**					
Primary education	128	1			
Secondary education	63	0.782	0.371	1.65	0.517
University/college	25	1.24	0.357	4.31	0.734
education					
No formal education	19	0.437	0.114	1.67	0.226
**Knowledge of risk factors**					
Yes	31	1			
No	199	1.497	0.233	9.61	0.67
**Knowledge of HIV+ve as risk factor**					
Yes	153	1			
No	77	0.492	0.253	0.96	0.037
**Knowledge of preventive measures**					
Yes	63	1			
No	170	0.114	0.013	0.97	0.047
**Parity**					
1 - 2 births	137	1			
3 or more births	68	2.124	1.012	4.46	0.046
None	30	0.999	0.358	2.78	0.999
**Employment status**					
Businesswoman	152	1			
Informal sector	40	0.557	0.207	1.5	0.247
Housewife	43	2.594	1.149	5.85	0.022
**Age at sex onset**					
10 - 19 years	17	1			
20 - 25 years	213	0.894	0.757	1.06	0.184
**Previous history of STDs**					
Yes	17	1			
No	213	0.554	0.172	1.79	0.322
**Family history of cervical cancer**					
Yes	21	1			
No	209	0.316	0.096	1.04	0.059

## Discussion

This study has found that poor number of women had screened for cervical cancer which is similar to reports from other low-middle income countries.^10^,^26^-^29^ While there are national and international guidelines on preventive measures for cervical cancer, utilisation of cervical cancer screening is low which suggests low level of awareness and understanding of the need to screen for cervical cancer among the studied population. Therefore, there could be other factors (e.g., social and cultural) deeply rooted in these communities which influence cervical cancer screening uptake that could not be determined quantitatively. With only nine years remaining before 2030, attainment of the WHO recommendation of 70% screening for cervical cancer is not likely to be achieved unless massive campaigns on cervical cancer screening are enhanced by governments in the Africa region.

We found poor association between screening for cervical cancer and younger ages, but the association increased with age. This finding is contrary to previous reports^30^,^31^ and explanation for the trend in this study could be that screening for cervical cancer is influenced by individual health seeing behaviour which can be promoted by information provided through media and health campaigns. In addition, the perceptions of being young and healthy, therefore, low risks for cervical could explain the poor screening rate among younger women. For the older ages, fear of diseases of old age, the likelihood to contract chronic diseases like cancers, and family history of cancers are likely to be important drivers for screening rates in the older age group.

We also found that the knowledge of HIV being a risk factors for cervical cancer influenced utilisation of cervical cancer screening services. This finding is supported by previous findings in Kilimanjaro, Tanzania and Cote d'Ivoire, West Africa.^31^,^24^ The association could be through increased risk perception of HIV and associated diseases which could be motivating individual women to pro-actively utilise screening services both for treatment and prevention.

Inadequate knowledge on the prevention of cervical cancer was found to influence utilisation of cervical cancer screening services. This finding suggests that information on cervical cancer prevention among women is low therefore, vigorous and integrated community health campaigns, if possible, with self-administered HPV screening is required to raise the level of knowledge on the prevention of cervical cancer and increase the screening services uptake. Community engagement and designing innovative strategies to complement existing strategies could yield more positive outcomes.

Parity showed to be a significant factor influencing utilisation of cervical cancer screening services and women with high parity of three and above were more likely to screen for cervical cancer possibly because of multiple exposure to information given to them at the CTC clinics on the association of HIV and cervical cancer, the need for screening and preventive measures. We however, found it interesting that the Odds of cervical cancer screening were lower among women with less parity because they are expected to be young, educated and likely to have access to correct information on the association of HIV and cervical cancer and preventive measures which is readily accessible from the internet, media and other sources. Similarly, the finding of higher odds for screening among housewives was not expected when compared to employed women who have been reported to be more likely to screen for cervical cancer.^32^ The busy schedules and pressure from work among employed women could be a result of challenges in finding time to screen for cervical cancer while support and encouragement from spouses and the ability to find time to attend to their health could explain the higher cervical cancer screening uptake among housewives. Therefore, campaigns and dissemination of correct information targeting most women on the potential risk factors and preventive measures should be intensified.

## Conclusion

Large population of HIV positive women are at risk of cervical cancer because they lack knowledge on HIV as a risk factor, and have inadequate knowledge on the prevention of cervical cancer. Parity and employment status are among the factors influencing utilisation of cervical cancer screening services. These findings suggest that provider-patient health education on screening for cervical cancer is needed to intensify utilisation of cervical cancer screening services among HIV positive women.

## Limitation of the study

The study has elucidated risks factors which may comprise the global initiative for cervical cancer elimination through 70-90-90 by 2030. However, the quantitative nature of the study has limitations to explain reasons of such factors and predictors on the uptake of cervical cancer screening. Qualitative studies are recommended in order to gathers participants' experiences, perceptions, and behaviour in order to answer such questions.
